# The influence of a supervised group exercise intervention combined with active lifestyle recommendations on breast cancer survivors’ health, physical functioning, and quality of life indices: study protocol for a randomized and controlled trial

**DOI:** 10.1186/s13063-021-05843-z

**Published:** 2021-12-18

**Authors:** Luiz Augusto Riani Costa, Raphael F. Barreto, Sarah Milani Moraes de Leandrini, Aline Rachel Bezerra Gurgel, Gabriel Toledo de Sales, Vanessa Azevedo Voltarelli, Gilberto de Castro, Sally A. M. Fenton, James E. Turner, Christian Klausener, Lucas Melo Neves, Carlos Ugrinowitsch, Jose Carlos Farah, Cláudia Lúcia de Moraes Forjaz, Christina May Moran Brito, Patricia Chakur Brum

**Affiliations:** 1 School of Physical Education and Sport, University of Sao Paulo, Av. Professor Mello Moraes, 65- Butantã, São Paulo, SP 05508-900 Brazil; 2 Instituto do Cancer do Estado de Sao Paulo, ICESP, Faculdade de Medicina da Universidade de Sao Paulo, Sao Paulo, Brazil; 3 School of Sport, Exercise and Rehabilitation Sciences, University of Birmingham, Birmingham, UK; 4 Department for Health, University of Bath, Bath, UK; 5 Centro de Práticas Esportivas da Universidade de Sao Paulo (CEPEUSP), Sao Paulo, Brazil; 6 Master’s Program in Health Sciences at Santo Amaro University – UNISA, Sao Paulo, Brazil; 7 Bipolar Disorder Program (PROMAN), Department of Psychiatry, University of São Paulo – USP, Sao Paulo, Brazil; 8 Departamento de Biodinamica do Movimento do Corpo Humano, Escola de Educação Física e Esporte da Universidade de São Paulo, Av. Professor Mello Moraes, 65- Butantã, São Paulo, SP 05508-900 Brazil

**Keywords:** Exercise, Breast neoplasms, Physical fitness, Motivation, Canoeing

## Abstract

**Background:**

Most cancer patients, under active treatment or not, are sedentary, despite increasing scientific and clinical understanding of the benefits of exercise and physical activity, such as improving quality of life, limiting disease symptoms, decreasing cancer recurrence, and increasing overall survival. Studies have shown that both supervised exercise and unsupervised physical activity programs have low adherence and limited long-term benefits among cancer survivors. Therefore, interventions focused on increasing physical activity levels have clinical and psychological relevance. The present study will examine the feasibility and efficacy of an intervention that combines supervised group exercise with active lifestyle recommendations, analyzing its clinical, psychological, physiological, functional, and immunological effects in breast cancer survivors.

**Methods:**

Women aged 35–75 years who have completed chemotherapy, radiotherapy, and surgery for breast cancer will be recruited from the Cancer Institute of the State of Sao Paulo (ICESP) and take part in a 16-week, parallel-group, randomized, and controlled trial. They will receive a booklet with recommendations for achieving a physically active lifestyle by increasing overall daily movement and undertaking at least 150 min/week of structured exercise. Then, they will be randomized into two groups: the supervised group will take part in two canoeing group exercise sessions every week, and the unsupervised group will increase their overall physical activity level by any means, such as active commuting, daily activities, or home-based exercise. Primary outcome includes aerobic capacity. Secondary outcomes are physical activity, physical functioning, self-reported quality of life, fatigue, presence of lymphedema, body composition, immune function, adherence to physical activity guidelines, and perceptions of self-image.

**Discussion:**

Results should contribute to advance knowledge on the impact of a supervised group exercise intervention to improve aspects related to health, physical functioning, and quality of life in female breast cancer survivors.

**Trial registration:**

Brazilian Registry of Clinical Trials Number: RBR-3fw9xf. Retrospectively Registered on 27 December 2018. Items from the World Health Organization Trial Registration Data Set can be accessed on http://www.ensaiosclinicos.gov.br/rg/RBR-3fw9xf/.

**Supplementary Information:**

The online version contains supplementary material available at 10.1186/s13063-021-05843-z.

## Introduction

Advances in breast cancer diagnosis and therapy have improved the 5-year overall survival rates among these patients, now exceeding 90% in early stages [1–3]. These improved outcomes have called health professionals’ attention to those long-term side effects faced by breast cancer survivors either related to the disease or to their treatment [4–6]. As an example, a substantial fraction of breast cancer survivors does experience long-term side effects that include fatigue, lymphedema, peripheral neuropathy, neurocognitive dysfunction, and persistent pain, as well as an increased risk of developing cardiovascular disease, metabolic disturbances, and compromised mental health (e.g., depression and anxiety) [7–13]. All these aspects negatively affect quality of life of breast cancer survivors. Therefore, non-pharmacological therapies that aim to ameliorate these long-term consequences are critical to improving overall survival and health-related quality of life of breast cancer survivors and should be considered a public health priority. In this sense, the incorporation of healthy and active lifestyle that includes physical activity, weight management, self-care, adequate diet, and nutrition are encouraged [14–21].

The impact of both physical activity and exercise on breast cancer survivors is well documented. The term physical activity applies to any body movement produced by skeletal muscles that increase the energy expenditure, and exercise is considered as a structured physical activity that is undertaken with the purpose of improving health and fitness. In the context of cancer risk and survivorship, physical activity and exercise are linked to reduced risk of cancer recurrence [22, 23], secondary cancers [24], and cancer-specific cardiovascular mortality [25–29]. Indeed, systematic reviews and experimental research clearly describe the positive impact of physical activity and exercise on several aspects of physical and social well-being, and mental health among cancer patients under active treatment or not; or on cancer survivors, including reduced lymphedema, fatigue, depression, and anxiety, while improved cognitive function, cardiovascular fitness, self-esteem, and quality of life [30–36].

The convincing body of basic and clinical studies on the safety and benefits of exercise or physical activity in cancer patients does support the endorsement of post-treatment survivorship guidelines proposed by international organizations for both exercise (e.g., The American College of Sports Medicine) and cancer care (e.g., Clinical Oncology Society of Australia) [37–43]. Typically, guidelines advise people living with and beyond their cancer to engage in at least 150 min of moderate-to-vigorous physical activity per week, which can take the form of structured, purposeful exercise [14, 41, 43, 44]. Recent guidelines emphasize that moderate-to-vigorous exercise and physical activity can be achieved through a combination of aerobic, resistance and flexibility exercises, and supervised interventions are most effective [45–48].

However, despite consistent evidence that regular structured exercise can help cancer patients to better manage the acute- and long-term disease- and treatment-related effects on their health and well-being, research suggests up to 70% of cancer patients do not reach the recommended levels of moderate-to-vigorous physical activity [30, 49–52]. In fact, these patients undergo a sustained reduction in their physical activity levels following diagnosis, which can persist years after treatment remission [53–55]. Reasons cited for low engagement in physical activity include social and environmental barriers to engaging in supervised exercise, such as transportation costs and accessibility, financial problems, time, and distance to a training facility. To address such barriers, home-based unsupervised physical activity programs have been proposed with guidance provided by instruction booklets [56–61]. However, even though home-based programs offer a more flexible, feasible, and accessible option for increasing physical activity levels among cancer patients, poor uptake and low adherence remain a challenge [42, 62–67]. Therefore, alternative interventions that have the potential to engage and motivate people living with cancer to adopt and maintain an active lifestyle are critical. In this sense, group-based exercise interventions seem to facilitate greater improvements in overall quality of life when compared with personal training intervention [68]. However, further studies are needed to clarify the effects of a group-based exercise for encouraging incorporation of an active lifestyle and improving health and physical functioning in cancer survivors.

Based on current strategies to encourage exercise engagement and referral as regular practice for cancer survivors [69–73], we designed a randomized trial to test the impact of a supervised group exercise intervention combined with booklet recommendations compared with unsupervised physical activity at home following booklet recommendations among breast cancer survivors. We hypothesize that exercise supervision in a group model is a key parameter for encouraging both the adoption and adherence to exercise, and thus sustaining an active lifestyle leading to improvements in global health, physical functioning, and quality of life.

## Study aim and objectives

The aim of the study is to assess the impact of a 16-week supervised group exercise intervention, combined with physical activity recommendations delivered via a booklet, on overall physical activity, physical functioning, and quality of life among breast cancer survivors. The primary outcome includes aerobic capacity (assessed via directly measured peak oxygen consumption, or VO_2_)_._ Secondary outcomes are physical activity (assessed via accelerometry and questionnaire), physical functioning (assessed via muscle strength, balance, and agility), self-reported quality of life, fatigue, presence of lymphedema, body composition, immune function, adherence to physical activity guidelines, and perceptions of self-image.

Additionally, we will investigate whether supervised aspects of the intervention enhanced participants’ motivation for physical activity and exercise, and whether changes in motivation are linked to adherence to exercise program and higher levels of physical activity overall.

## Methods

### Study design

**Fig. 1:**
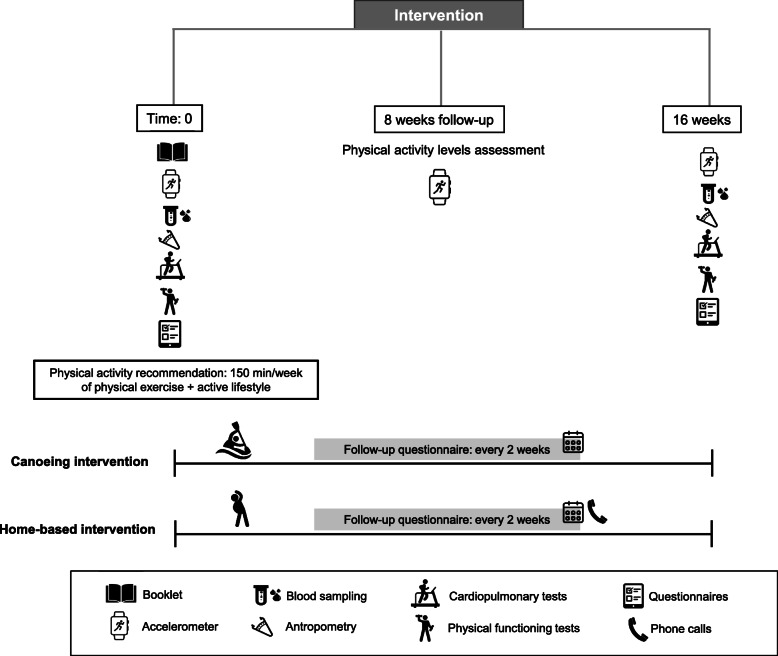
Overall design of the study

The present study is a prospective, longitudinal, pre-post-intervention (Fig. 1), open-label, active-controlled randomized superiority trial with participants allocated into two parallel-groups:

1. Supervised group: canoeing and physical activity recommendations
2. Unsupervised group: home-based physical activity recommendations

Our hypothesis is that the supervised group exercise intervention combined with physical activity recommendations will achieve better adherence to active lifestyle, exhibiting greater improvements in global health, physical functioning, and quality of life than the unsupervised intervention. Both groups will receive advice on adopting an active lifestyle, based on standard physical activity recommendations established by the World Health Organization (WHO). The advice will be provided verbally in a meeting (see section “Participant randomization and group allocation”) and via a booklet containing physical activity recommendations produced by the Cancer Institute of the State of Sao Paulo (ICESP). Both groups will be advised on increasing general daily movement (e.g., commuting, home-based activities, and leisure time physical activity) and undertaking at least 150 min per week of structured exercise (i.e., aerobic, strength, or flexibility), trying to exceed these recommendations by any movement including physical therapy, gym, sports, structured exercises, and not structured physical activity, discriminating the activities with specific questionnaires applied every 2 weeks during intervention. Participants in the supervised group will take part in group canoeing training sessions twice a week, while the unsupervised group will engage in physical activity and exercise of their choice. Considering the standard delivery of physical activity recommendations adopted in cancer centers to promote an active lifestyle, the unsupervised group is taken as the control group in this study. Adherence to the WHO physical activity recommendations outlined in the booklet will be monitored every 2 weeks throughout the 16-week intervention period in both groups via a standardized questionnaire, which will be completed in-person by participants in the supervised group and via telephone call in the unsupervised group. The findings from this study will be reported in accordance with the recommendations of the Consolidated Standards of Reporting Trials (CONSORT) guidelines as described in the section “Discussion”.

### Participant recruitment and eligibility criteria

Participants will be recruited at ICESP. A researcher (RB) will access medical records and will contact eligible patients who meet the inclusion criteria: histologically proven invasive breast cancer; absence of distant metastases (as documented by the local recommendations of staging work-up); patients who have finished the prescribed breast cancer treatment with curative intent, including surgery (either breast conserving surgery or mastectomy), systemic cytotoxic chemotherapy (following local guidelines, including adriamycin- and/or taxane-based regimens, as in adjuvant and/or neoadjuvant settings), and radiotherapy; aged between 35 and 75 years old, and have concluded their treatment within a time span of at least 6 months up to 3 years. Active endocrine treatment will be allowed. Eligible participants will be contacted by the researcher via telephone, receive explanations on the study, and then, asked whether they would like to take part. For willing participants, physicians of the Cancer Institute will evaluate their medical records and, if feasible, give medical authorization for participation on the trial. Exclusion criteria include participants who present 1) evidence of metastatic disease (clinical and/or radiological); 2) severe lymphedema as judged by the investigators; 3) severe organic dysfunctions (including, but not restricted to kidney failure, heart failure, and chronic liver disease); 4) uncontrolled hypercholesterolemia, diabetes, and/or hypertension; 5) any medical contraindication (including, but not restricted to, severe pain, musculoskeletal lesion, cardiovascular, or metabolic risk), or 6) have been engaged in regular physical training for the past 4 months. To be eligible to canoeing exercise, group participants must be able to swim 50 m in a swimming pool using a life jacket as assessed by a member of the research team. Only participants who remain calm in water will be recruited.

### Ethical compliance

**Fig. 2:**
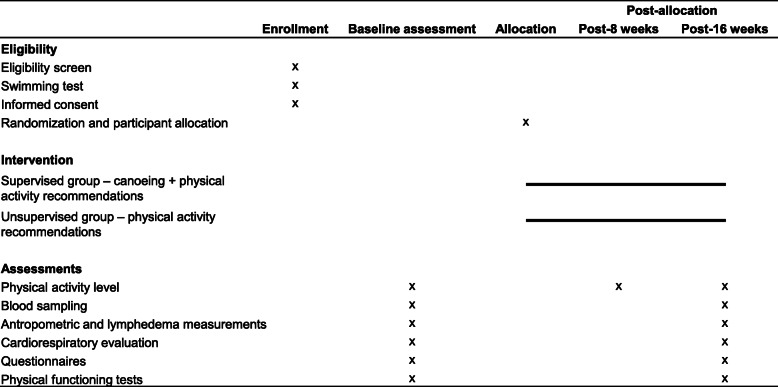
Overall schedule of enrollment, intervention, and assessments following the Standard Protocol Items: Recommendations for Interventional Trials (SPIRIT)

This study protocol is registered in a database of clinical research studies (Brazilian Registry of Clinical Trials - ReBec) under the number RBR-3fw9xf and was approved by local Ethical Committees (Research Ethics Committee of the USP School of Physical Education and Sports approval: 2.441.435, and Commission of Ethics in Research in Human Beings of the USP Medical School approval: 2.836.680). This manuscript is described according to the Standard Protocol Items: Recommendations for Interventional Trials (SPIRIT) checklist (Fig. 2, Additional file 1) and the study is performed in accordance with the ethical principles laid down in the seventh and current edition (2013) of the Declaration of Helsinki.

Participants who meet the eligibility criteria and have been authorized for participation will receive a detailed explanation about the protocol and provide written consent, including storage of biological specimens. Participants will be informed that they can withdraw from the study at any time without consequence. The intervention will be discontinued for the participants who 1) request to stop, 2) have any surgery scheduled, or 3) suffer any injuries that prevent the practice of physical activity or exercise. The reasons for drop-out will be recorded and all data collected from these participants will be included in the analysis. From the time point of discontinuation, no outcome will be collected from those participants. The protocol poses a low risk to the participants regarding blood sampling and cardiorespiratory evaluation. Both procedures will be performed by an experienced physician (LARC) and participants will be monitored after the cardiorespiratory fitness test until they fully recover from the exercise bout. During the canoeing or exercise training sessions, participants will always wear life jackets, and if they feel any discomfort, they will receive local medical care and will be taken to the nearby University Hospital if needed. Likewise, all participants will continue their routine medical care at ICESP.

### Participant randomization and group allocation

Prior to group randomization, a researcher blinded to the future group allocation will classify participants into quartiles based on their peak VO_2_ values. Subsequently, participants from each quartile will be randomly assigned with a 1:1 allocation ratio to 1) supervised group: canoeing and physical activity recommendations (*n* = 18), or 2) unsupervised group: physical activity recommendations (*n* = 18). Following randomization, participants in both groups will be invited to a meeting at the School of Physical Education and Sport at the University of Sao Paulo, where the researcher and the physician will communicate the importance of an active lifestyle and the benefits of physical activity for people living with cancer. In addition, participants will be introduced to the physical activity recommendations booklet (based on WHO guidelines) produced by ICESP. The researcher and physician will outline the goal of achieving physical activity guidelines (i.e., 150 min of moderate-to-vigorous physical activity/exercise per week). Participants will be advised that recommendations can be achieved through separate bouts of activity, such as 50 min per day on three non-consecutive days each week, or 30 min 5 days a week. Following, the physician, who will be blinded to the allocation, will leave the room and the researcher will inform each participant which intervention group they have been allocated to. For participants in the supervised group (canoeing and physical activity recommendations), canoeing training schedules are explained, whereas for those participants allocated in the unsupervised group, best days and times for telephone calls will be arranged so the researcher can remotely investigate whether this group is reaching physical activity recommendations.

### Physical activity interventions

#### Supervised group—canoeing and physical activity recommendations

Participants will take part in canoeing sessions twice a week at the Olympic Lane of the Sports Centre of the University of Sao Paulo, under the supervision of an experienced researcher and instructors (RB, CK, and JCF). Training sessions will consist of a warm-up followed by the main training session (canoeing exercise), which will last approximately 40 min long, and a cool-down phase. Weeks 1 and 2 are meant to familiarization with the practice of canoeing, which is performed in continuous strokes; also, to familiarization with data collection methods (e.g., individual monitoring of heart rate and effort perception sampled every 5 min during the 30 min session). Training intensity will be increased every 2 weeks as follows: weeks 1 and 2: 35 rows/min, weeks 3 and 4: 40 rows/min, weeks 5 and 6: 45 rows/min, weeks 7 and 8: 50 rows/min, weeks 9 and 10: 55 rows/min, weeks 11 and 12: 60 rows/min, weeks 13 and 14: 62 rows/min, and weeks 15 and 16: 65 rows/min. In addition, high-intensity interval training will be introduced in the last 15 min of each session, by increasing row frequency in series. Variables indicating the intensity of individual and group effort will also be measured in all training sessions, namely: heart rate, ratings of perceived exertion via the Borg scale, row frequency, speed, distance covered, time of each activity, and total training time. Distance covered will span from 1.6 to 2.1 km as familiarization with canoeing practice and endurance increase overtime. Participant’s adherence to the canoeing intervention will be measured by recording attendance at training. The participants will also be constantly encouraged by the researcher to engage in physical activity beyond the supervised canoeing training, to achieve the recommended 150 min of moderate-to-vigorous physical activity weekly.

#### Unsupervised group—physical activity recommendations

Participants will receive physical activity recommendations booklet (based on WHO guidelines) produced by ICESP. The booklet suggests different types of physical activity and exercise, such as aerobic, strength, and flexibility, outlines their benefits, and precautions participants should adopt while exercising. The booklet recommends exercise as part of a lifestyle behavioral change. Participants will be free to perform daily life activities and physical activity at home, at gyms or outdoors, with the aim of increasing overall physical activity including leisure time physical activity via structured exercise. A member of the research team will make telephone calls to the participants, which are scheduled to be done every 2 weeks, totalizing eight phone calls. In these calls, the researcher will apply a standardized follow-up questionnaire to monitor physical activity adherence.

### Assessments

**Fig. 3:**
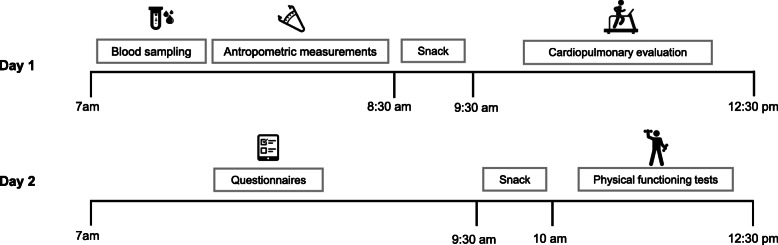
Schematic overview of the main trial data collection days; lines indicate timing of blood sampling, anthropometric measurement collection, cardiorespiratory test, questionnaires, and physical functioning analysis, over two visits

The experimental procedures described below will be performed at baseline (Pre) and after 16 weeks of the intervention (Post), keeping an interval of 3 or 4 days after the last canoeing training session. An exception will be the evaluation of physical activity engagement (time, frequency, and intensity), which will also be monitored at 8 weeks (mid-way point of the intervention). The assessment protocol will be divided into two visits and all procedures will take place at the School of Physical Education and Sport of the University of Sao Paulo (Fig. 3). Participants will be advised to avoid performing physical activities and alcohol ingestion in the 24 h prior to the visits, to refrain from tobacco use and stimulant beverages (coffee, tea) for at least 6 h, and to take their medication as recommended by their physicians. In the first visit, the participants will arrive at 7 am, following an 8-h fasting period and undertake the procedures in the following order: 1) blood sampling; 2) anthropometry and lymphedema measurement; 3) eat a snack consisting of an apple, 150 g of salted crackers, and 200 ml of juice; and 4) cardiorespiratory fitness test. The second visit will take place at least 2 days later to allow the participant to fully recover from the fitness test. For the second visit, the participants will arrive at 7 am and undertake the following evaluation: 1) questionnaires assessing quality of life in cancer survivors, health status, fatigue, body image, and physical activity motivation; and 2) physical functioning tests.

#### Blood sampling

Blood samples will be collected by puncture of a forearm peripheral vein by an experienced physician (LARC). If the participant had undergone the removal of axillary lymph nodes, the blood will be taken from the unaffected arm. Blood samples will be collected into two 4-ml BD Vacutainer® tubes coated with dipotassium ethylenediaminetetraacetic acid (K_2_EDTA) and one 5 ml BD Vacutainer® SST® tube (with silica clot activator, polymer gel, silicon-coated interior). The gel tube will be centrifuged at 2100*g* for 10 min at 4 °C to isolate the plasma and together with the K_2_EDTA tubes will be sent to the Clinical Hospital Central Laboratory (School of Medicine, University of Sao Paulo) for standard biochemical and immunological analyses. The laboratory analysis of the blood samples will include the following: complete blood and platelet count assessed using an automated analyzer followed by microscope revision; glycated hemoglobin assessed by high-performance liquid chromatography (HPLC); total cholesterol, high-density lipoprotein (HDL), non-HDL and low-density lipoprotein (LDL) cholesterol assessed using enzymatic colorimetric cholesterol oxidase phenol 4-aminoantipyrine peroxidase Isotope Dilution - Mass Spectrometry (CHOD/PAP ID-MS) assays; triglycerides assessed using enzymatic colorimetric Glycerol Phosphate Oxidase Peroxidase Amidopyrine (GPO-PAP) assay; and glucose assessed using ultraviolet (UV) enzymatic method with hexoquinase. The remaining plasma from the chemistry analysis will be stored at − 80 °C for future molecular analysis within the research objectives. An additional 25 ml of whole blood will be collected into sodium heparin BD Vacutainer® tubes and peripheral blood mononuclear cells (PBMCs) will be isolated using density gradient centrifugation using standard methods. Cells in freezing medium [70% Roswell Park Memorial Institute (RPMI) media, 20% fetal bovine serum (FBS), 10% dimethylsulphoxide (DMSO)] will be cryopreserved at − 1 °C/min using a Nalgene “Mr Frosty” container in a − 80 °C freezer and after 24–48 h will be moved to long-term storage in liquid nitrogen at − 196 °C. For immune function assays, cells will be thawed in batches, rested overnight, and examined using enzyme-linked immunospot (ELISPOT) assays. Briefly, the magnitude of interferon gamma (IFN-γ) production by T cells will be assessed in response to a variety of stimuli, including antigens from common viruses such as *Cytomegalovirus*, *Epstein Barr Virus*, *Varicella Zoster Virus*, and tumor-associated antigens pre- and post-intervention.

#### Anthropometry and lymphedema measurement

Body mass will be assessed using digital scales, and height measurements performed using a wall-mounted stadiometer, with the participant standing upright and barefoot. Body mass index (BMI) will be calculated by the measures of weight (W, kg) and height (H, m) using the equation BMI = W / H^2^. Waist circumference will be measured using a measuring tape placed around the smallest abdominal diameter. Body composition will be estimated by skinfold thickness from triceps, suprailiac, and medial thigh using an adipometer. The following protocol will be employed for conversion of body density [74]:

• % Fat (Siri’s equation) = [(4.95 / DENS) − 4.50] × 100.

• DENS = 1.099421 − 0.0009929 (X2) + 0.0000023 (X2) 2 − 0.0001392 (X3)

where X2 = sum of the skinfolds: triceps, suprailiac, and thigh; and X3 = participant’s age, in years.

Lymphedema status will be assessed by measuring the bicipital circumference. Participants will adopt the orthostatic position, with arms on the anatomical position, extended along the body. A measuring tape will be placed around the midpoint of the arm.

#### Cardiorespiratory fitness test evaluation

The laboratory temperature will be maintained between 20 °C and 22 °C. The test will be conducted on a treadmill (Inbrasport®, RS, Brazil) at least 60 min after participants have consumed the standard snack (as described previously). The test will begin with a 5-min walking at 2.0 mph to analyze walking economy. Afterwards, treadmill speed will be increased by 0.5 mph each minute until the participant achieves a maximal comfortable speed (3.0 mph, 3.4 mph, or 4.0 mph) previously defined in an adaptation test. Then, the treadmill grade will be increased by 2% each minute (Table 1) until the participants can no longer continue. Electrocardiogram will be continuously monitored (EMG System do Brazil, EMG, 030110/00B, Sao Paulo, Brazil) throughout the test and auscultatory blood pressure will be measured using a mercury column sphygmomanometer (Uniteq) at the end of the 5 min walking period and every 2 min during the progressive test. Oxygen consumption (VO_2_) will be measured with breath-by-breath sampling using a metabolic cart (CPX Ultima, Medical Graphics Corporation® CPET system, St. Paul, MN) with data analyzed by 30 s averages. Anaerobic threshold and respiratory compensation point will be determined in accordance with Skinner and McLellan’s criteria [75] by two researchers, with a third researcher consulted to solve discrepancies. Peak heart rate and peak VO_2_ will be recorded at each participant’s exhaustion.

**Table 1:** Progressive exercise test protocol

	Target speed 3.0 mph		Target speed 3.4 mph		Target speed 4.0 mph	
Time	Speed	Grade	Speed	Grade	Speed	Grade
5 min	2.0 mph	0%	2.0 mph	0%	2.0 mph	0%
6 min	2.5 mph	0%	2.5 mph	0%	2.5 mph	0%
7 min	3.0 mph	0%	3.0 mph	0%	3.0 mph	0%
8 min	3.0 mph	2%	3.4 mph	0%	3.5 mph	0%
9 min	3.0 mph	4%	3.4 mph	2%	4.0 mph	0%
10 min	3.0 mph	6%	3.4 mph	4%	4.0 mph	2%
11 min	3.0 mph	8%	3.4 mph	6%	4.0 mph	4%
12 min	3.0 mph	10%	3.4 mph	8%	4.0 mph	6%
13 min	3.0 mph	12%	3.4 mph	10%	4.0 mph	8%
14 min	3.0 mph	14%	3.4 mph	12%	4.0 mph	10%

#### Questionnaires (pre- and post-intervention)

The questionnaires will be administered to participants by a trained researcher. To guide participants in completing the questionnaire, the researcher will explain each questionnaire and read each item aloud, encouraging participants to ask any question they may have. The following questionnaires will be administered:

i. European Organization for Research and Treatment for Cancer Quality of Life Questionnaire - Core 30 (EORTC QLQ-C30) will be used to assess a broader dimension of quality of life in cancer survivors [76]. This validated instrument is composed of three scales that examine global health, functional aspects and patient’s symptoms. It comprises 28 questions about difficulties of daily life and symptoms with scales from 1 (no difficulty/distress/symptom) to 4 (a lot of difficulty/distress/symptom), and two questions about general health and quality of life with scales from 1 (terrible quality) to 7 (great quality). The Brazilian version has been previously validated among people living with cancer [77].
ii. 36-Item Short-Form Health Survey (SF-36) will be used to evaluate health status [78]. It comprises eleven questions (five of which are multi-item) that evaluate health status including physical and social functioning, physical and emotional role, mental health, bodily pain, general health perceptions, and vitality. The Brazilian version of SF-36 was already applied to women with a history of breast cancer [79].
iii. Piper Fatigue Scale-Revised (R-PFS) will be used as a subjective fatigue assessment method. Its psychometric properties have been validated in female survivors of breast cancer [80]. The Brazilian version contains 22 items on three dimensions (behavioral, affective, and sensorial–psychological) and provides an overall of fatigue score on a scale from 0 (the best status evaluation) to 10 (the worst status evaluation). This instrument has been validated for oncological use in Brazilian populations [81], particularly for breast cancer survivors [82].
iv. Body Image Scale [83] will be used to assess body image. Its higher scores indicate greater body appreciation and it addresses six domains of body image: vulnerability, body stigma, limitations, body concerns, transparency, and arm concerns. The Brazilian version presents 44 statements for which the participant must decide how they apply to her, on a scale varying from 1 (Strongly disagree/Never/Almost Never) to 5 (Strongly Agree/Always/Almost Always). It has also been demonstrated to be reliable and valid [84].
v. Inventory of Motivation to the Regular Practice of Physical Activity (IMPRAF-54, Brazilian acronym) will be used to assess physical activity motivation. The short version of this form contains 54 items for which the participant must decide how they reflect her motivation to engage on physical activity, using a scale varying from 1 (this motivates me very little) to 5 (this motivates me a lot). All affirmations start with “I engage in physical activity to...” followed by sentences that reflect six dimensions (nine items for each), such as control of stress (e.g., decrease irritation), health (e.g., acquire health), sociability (e.g., meet friends), competitiveness (e.g., be champion in sport), esthetic (e.g., accomplish a beautiful body), and pleasure (e.g., achieve my ideals) [85]. The raw score obtained in each dimension will be related to a percentile according to participant’s gender and age, which will provide the participants’ motivational profile.

#### Assessing physical activity every 2 weeks

Quantifying physical activity will be critical for indicating the level of adherence to the intervention and to assess whether the participants have moved towards adopting a more physically active lifestyle [86]. In addition to obtaining estimates of physical activity using accelerometry, a standardized questionnaire will be administered by a trained researcher in-person/face-to-face in the supervised canoeing group, and via telephone in the unsupervised group every 2 weeks. The periodic assessment and contact with the participants are also a key strategy to promote retention, especially in the home-based group. The questionnaire will be modified from the Brazilian version [87] of the Minnesota Questionnaire on Physical Activities, Sports and Leisure [88], which surveys time spent on various activities, both domestic and sports. This physical activity questionnaire will be used to measure 1) the time participants spent doing accumulated physical activities, including lifestyle physical activity and incidental movement, referred as “Unscheduled Movement”; 2) the time spent in physical activities specifically scheduled to meet the goal of 150 min of physical exercise per week; 3) and the total time spent sitting, whether in leisure activities (such as watching television and cell phones) or in work activities. Self-reported measures of physical activity and sedentary behavior have been criticized for low validity and reliability. As a result, caution is advised when relying on questionnaires to assess physical activity and sedentary behavior [89]. To address this issue, physical activity will also be assessed by accelerometer pre-intervention, at 8 weeks and post-intervention (16 weeks), aiming to provide a more objective estimate to compare and validate the follow-up physical activity and the sedentary behavior questionnaire.

#### Physical activity level

Time, intensity, and frequency of physical activity engagement will be assessed using ActiGraph GT9X accelerometers (ActiGraph Pensacola, USA) at pre-intervention (with aims to provide baseline comparisons), during the intervention (mid-way, approximately 8 weeks of intervention), and post-intervention (after 16 weeks). The ActiGraph GT9X is a small, light-weight accelerometer (19 g; 4.6 cm × 3.3 cm × 1.5 cm) capable of recording accelerations in the vertical (Y), horizontal right-left (X), and horizontal front-back (Z) axes to compute vector magnitude [VM = √(axisY^2^ + axisX^2^ + axisZ^2^)]. The ActiGraph GT9X records accelerations over pre-defined time periods (“epochs”). Following which, Actilife software (ActiGraph Pensacola, USA) is used to convert raw acceleration data into “activity counts” for analysis. In this study, the GT9X+ will be initialized to record accelerations in 60-s epochs over a period of 7 days at each assessment time point. During this 7 days, participants will be asked to wear the GT9X attached to an adjustable elastic belt on their right hip in a vertical position [90, 91], and to remove the device only for sleeping and water-based activities (e.g., swimming, bathing).

##### Data reduction

Following 7 days wear*,* data will be downloaded using Actilife and cleaned to check for periods of non-wear. Non-wear criteria will be ≥ 60 min of consecutive “0” counts, with a spike tolerance of 2 min [91]. For accelerometer data to be considered valid and retained for use in subsequent analysis, participants will be required to have worn the accelerometer for ≥ 10 h/day, on ≥ 4 weekdays, including ≥ 1 weekend day [91]. Sedentary time as well as time, frequency, and intensity of PA will be estimated by applying count-based vector magnitude (VM) cut-points to valid accelerometer data. Cut-points that will be applied were selected based on widespread use in epidemiological research using the ActiGraph [91] and are considered in counts/min as sedentary time = < 100, light-intensity PA = 101–2019, moderate-to-vigorous intensity PA = ≥ 2020. The average daily time spent sedentary, in light and moderate-to-vigorous intensity (min/day), as well as the average daily proportion of time spent in these behaviors (i.e., % wear time = behavior [min/day]/total GT9X+ wear time [min/day]) × 100) will be computed for analysis.

#### Physical functioning tests

Participants will undertake a circuit of six functional fitness tests, designed to systematically address parameters associated with independent functioning: aerobic endurance, upper and lower body strength and flexibility, and agility/dynamic balance.

i. *Aerobic endurance*: Two-minute step test will be used to assess gait functionality. This test correlates highly with peak oxygen consumption, the primary outcome of this study [92].
ii. *Upper body muscular strength*: (i) Maximal isometric handgrip test (Jamar®) will evaluate hand muscle strength during the handgrip test, three alternating attempts will be made for each arm and the highest score will be used for analysis [62]; (ii) Arm curl test will be used to assess arm muscle strength. Participants will do forearm flexions during 30 s with a 2-kg weight. Three alternating attempts will be made for each arm, the first attempt will be considered a familiarization, and the mean of the last two curls will be used for analysis [92].
iii. *Lower body muscular strength*: Chair stand test will be used to determine lower limb strength. This test comprises sitting and standing repeatedly without the aid of the arms, for 30 s. Participants will attempt the test once and their score will be used for analysis [62].
iv. *Upper body flexibility*: Back scratch test will be used to measure general shoulder range of motion (flexion and extension) in the standing position. Three alternating attempts will be made for each arm. The first attempt will be considered a familiarization and the average of the last two attempts will be used for analysis [92].
v. *Lower body flexibility*: Sit and reach lumbar flexibility test will evaluate lower body range of motion using sit and reach bench. Three attempts will be executed. The first attempt will be considered a familiarization and the average of the last two attempts will be used for analysis [93].
vi. *Agility/dynamic balance*: Timed up-and-go test will be used to assess functional mobility as the time needed for the participant to get up from a chair, walk 3 m, and return to the original position—Two attempts will be made and the shortest time between them will be used for analysis [94].

### Statistical analysis

#### Power calculation and sample size

Sample size calculations were performed based on the predicted maximum oxygen consumption (VO_2_—ml/kg min) of patients with breast cancer [95] using G-Power Software (Version 3.1.9.2). Sample size was carried out a priori, assuming a power (1 − *β* error) of .90 and assuming *α* error of .05. The calculation was based on an F test with analysis of variance by repeated measures and between- and within-group effects. To eliminate possible sample size bias, the Hedges *g* calculation was used. The effect size used in the calculation was based on the work of Courneya et al. [95] considering the maximum oxygen consumption (VO_2_—ml/kg min) of patients with breast cancer. A pre- to post-test correlation value of 0.50 was assumed. Power calculations indicated a sample size of *n* = 15 participants per group were required to achieve a power = .90 and *α* error = .05. To account for participant drop-out during the intervention, the sample size was increased by 20% to *n* = 18 participants per group. Therefore, we aim to recruit a total sample of *n* = 36 participants to the study.

#### Data analysis

The data analyst will receive the raw data coded for participant names and group allocation after completion of data collection. Group allocation will be unblinded after termination of data analyses. The Shapiro–Wilk and Levene’s tests will be used to determine normality and variance equality, respectively, and non-normal scale data will be log transformed. If the transformation does not produce a normal distribution, residual analyses will be performed to test if influential points change the interpretation of the results. If interpretations change, these points will be manually removed. Linear mixed models having group and time as fixed factors and participants as a random factor will be performed for each dependent variable [maximal aerobic capacity (VO_2_ max), daily physical activity, and adherence to physical activity guidelines (assessed via accelerometry and questionnaire), physical functioning (assessed via muscle strength, balance, and agility), self-reported quality of life, fatigue and perceptions of self-image (assessed via specific validated questionnaires), presence of lymphedema, and body composition and immune function]. Baseline differences in outcomes will be examined by independent *t*-test. An analysis of covariance (ANCOVA) will be implemented including the baseline variable as a covariate, group as a fixed factor, and participants as a random factor, in case of differences in baseline values between groups. An intention-to-treat analysis will also be performed in case of a high incidence of missing values, using linear mixed models; the first analysis will be performed removing the missing values, while the second analysis will be performed incorporating the missing data points to determine the impact of missing values in the overall results [96]. Whenever a significant F-value is obtained, a post hoc test with Tukey’s adjustment will be performed. Ordinal data will also be analyzed using mixed models having group and time as fixed factors, and participants as random factor. Nominal data will be compared using chi-square distributions and significance will be determined by residual analyses (< − 2 or > 2).

#### Data management

Participant files will be stored in numerical order and stored in a secure and accessible place and manner for a period of 3 years after completion of the study. Data integrity will be enforced through a variety of mechanisms: referential data rules, valid values, range checks, and consistency checks against data already stored in the database (i.e., longitudinal checks) will be supported. Additional errors will be detected by programs designed to detect missing data or specific errors in the data. A full backup of the primary database will be performed daily by the cloud storage service (https://uspdigital.usp.br/repositorio/). Auditing trial will be monthly conducted by the PI (PCB) and the responsible for data verification (RB, LARC, and SMML).

## Discussion

Over the past two decades, growing scientific evidence has provided support for the efficacy of exercise to improve cancer-related health outcomes. Exercise is a beneficial adjunct therapy for improving treatment tolerance [73, 97, 98] and helps to mitigate patient’s symptom-reported end points as fatigue, pain, and depression [8, 30, 32–34, 44, 45, 50, 62, 99]. However, adoption and adherence to exercise programs among cancer survivors is low [53, 61, 66, 100]. Reasons for a lack of regular exercise among people living with and beyond cancer are multifactorial and involve social and demographic issues (i.e., cost, lack of facilities tailored for cancer survivors near home, age, socioeconomic and educational level), long-term effects of the cancer therapy (i.e., fatigue, compromised immune system, physical deconditioning), lack of motivation and discipline for individualized exercise programs, and a lack of recommendation by oncologists and other health professionals [21, 46, 50, 52, 63, 69].

**Fig. 4:**
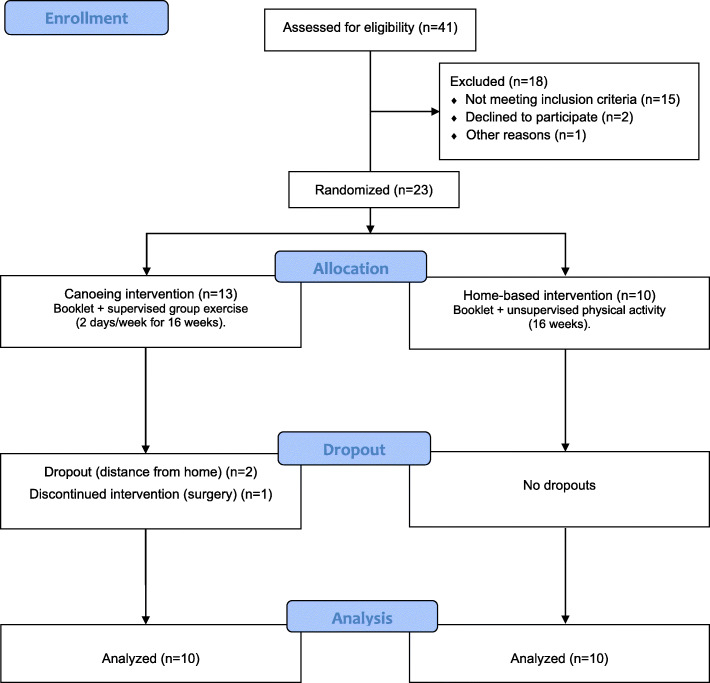
Consolidated Standards of Reporting Trials (CONSORT) flow diagram summarizing the trial status

The present trial will determine whether a supervised group exercise intervention combined to active lifestyle recommendations is a feasible option for breast cancer survivors. We expect that supervised exercise and social interaction offered by the group training sessions will be powerful strategies to improve exercise adherence, fitness, and socialization, offering an opportunity to participants to share experiences and enjoy mutual health-related improvements. We anticipate the supervised exercise group will present superior health, physical functioning, and quality of life indices after the intervention compared to the group receiving physical activity recommendations alone. The improvements we expect participants to experience following the supervised group-based exercise intervention may inspire participants to adhere to a long-term active lifestyle. The outcomes of this manuscript will be reported accordingly to the CONSORT guidelines (Fig. 4).

Prior to commencing the main trial, we conducted a small-scale pilot study to examine potential barriers to recruitment and the feasibility of protocol delivery. From this study, we learned that most of the participants in the unsupervised physical activity recommendations group did not answer phone calls during the intervention. Likewise, we acknowledge that some participants of this study neither did not have access to the ICESP booklet nor remembered to follow it, even though it is commonly used as a guideline by the Cancer Institute. Thus, to minimize these issues, we amended the protocol to include a standard talk about the benefits of physical activity for people living with cancer, immediately after the randomization. Furthermore, following the pilot study, we included the assessment of physical activity via accelerometry at the mid-intervention point (week 8), to provide a greater insight into change in physical activity behavior during the intervention. These procedures are meant to promote retention and complete follow-up of the participants.

Recruitment for the main trail has now commenced, and barriers identified to date include participants not found or not willing to take part, distance from the canoe training center (too far to travel), limitations imposed by the conventional therapy, movement restriction, and clinical clearance, among others. In addition, some barriers are observed once participants are recruited to the intervention, including requirement for mammary reconstruction surgery and distance from the canoe training center, which impact participants’ attendance to supervised exercise sessions. Regarding these barriers, the study staff will use all plausible means to overcome the limitations and support willing participants to continue in the study.

It is worth mentioning that after finishing the intervention period and concluding the assessments, all participants from both groups are advised to continue following an active lifestyle. As an encouragement, they are invited to enroll a Dragon Boat group training program offered at the Olympic Lane of Sports Center of the University of Sao Paulo at no cost. This is an integrated multidisciplinary program and partnership among ICESP, the Sports Center (Olympic Lane), and the School of Physical Education and Sport of the University of Sao Paulo. Cancer Institute’s physiatrists and oncology clinicians make triage and referrals, as well as nurses and physical therapists give advice and booklet recommendations for an active lifestyle. At the University of São Paulo’s Olympic Lane, participants exercise under supervision of physical education professionals and students. We assure that sustainable increase in exercise among cancer survivors relies on a qualified and collaborative multidisciplinary team that recognizes exercise as a safe and efficacious complementary therapy on cancer survivorship.

Our results will generate a body of evidence with the potential to advance knowledge on the impact of a supervised group exercise intervention to improve aspects related to health- and mental-related quality of life in female breast cancer survivors.

## Trial status

Recruitment began in June 2018. Initially, we would expect to conclude participants’ recruitment by July 2021. However, because of the current pandemic, recruitment should be delayed. This is version 3 of the protocol. Version 1 was submitted to the Research Ethics Committee of the USP School of Physical Education and Sports on November 2017; Version 2 included the ICESP as a co-participant institution and changed the age interval inclusion criteria from 45–75 to 35–75 years. Changes in version 3 were the exclusion of pain as an outcome (and the questionnaire to assess it), removal of obesity from the exclusion criteria, and the ones after the pilot study described in the “Discussion”.

## Supplementary Information

**Additional file 1.**

**Additional file 2.**

**Additional file 3.**

**Additional file 4.**

**Additional file 5.**

**Additional file 6.**

**Additional file 7.**

## Data Availability

The data generated and/or analyzed during the current study will be entered in duplicate under responsibility of RB, LARC, and SMML and organized in datasheets (Microsoft Excel files). Data from the pre and post assessments (laboratory analysis of blood samples, anthropometry and lymphedema measurement, cardiorespiratory fitness test evaluation, questionnaires, physical functioning tests, and physical activity levels via accelerometry) will be entered after the data collection days. Data from the questionnaire to assess physical activity and from the canoeing training sessions will be entered every 2 weeks. All data in paper format will be stored in a locked storage under the PI responsibility. Digital data will be kept in hard and online drives, saved, and kept on an institutional secure locked cloud (Google Drive) under protection and responsibility of researchers and available in the intranet repository from the University of Sao Paulo (https://uspdigital.usp.br/repositorio/). Final data produced by this study will be compiled in the abovementioned cloud computing system, which will be appropriately password protected. The datasets of the current study will be available from the corresponding author on reasonable request. The results of the protocol will be available to the public through communication channels of the School of Physical Education and Sport and ICESP.

## Abbreviations

ANCOVA: Analysis of covariance
BMI: Body mass index
CHOD/PAP: Cholesterol oxidase phenol 4-aminoantipyrine peroxidase
CONSORT: Consolidated Standards of Reporting Trials
DMSO: Dimethylsulphoxide
ELISpot: Enzyme-linked immunospot
EORTC QLQ-C30: European Organization for Research and Treatment for Cancer Quality of Life Questionnaire-Core
FBS: Fetal bovine serum
GPO-PAP: Glycerol phosphate oxidase peroxidase amidopyrine
H: Height
HDL: High-density lipoprotein
HPLC: High-performance liquid chromatography
ID-MS: Isotope Dilution-Mass Spectrometry
IMPRAF-54: Inventory of Motivation to the Regular Practice of Physical Activity
IFN-γ: Interferon Gamma
K_2_EDTA: Dipotassium Ethylenediaminetetraacetic Acid
LDL: Low-density lipoprotein
PBMCs: Peripheral blood mononuclear cells
R-PFS: Piper Fatigue Scale-Revised
RPMI: Roswell Park Memorial Institute media
SF-36: 36-Item Short-Form Health Survey
SPIRIT: Standard Protocol Items: Recommendations for Interventional Trials
UV: Ultraviolet
VM: Vector magnitude
VO_2_: Oxygen consumption
W: Weight
WHO: World Health Organization
