# Supplementary material for: The influence of a supervised group exercise intervention combined with active lifestyle recommendations on breast cancer survivors’ health, physical functioning, and quality of life indices: study protocol for a randomized and controlled trial
Source: Trials. 2021 Dec 18;22:934. doi: 10.1186/s13063-021-05843-z (PMC8684206; doi:10.1186/s13063-021-05843-z)
Supplement: Supplementary file 2 — Additional file 2. [file 13063_2021_5843_MOESM2_ESM.pdf]

PARECER CONSUBSTANCIADO DO CEP

DADOS DO PROJETO DE PESQUISA

Título da Pesquisa: EFEITOS DE DIFERENTES MODALIDADES DE ATIVIDADE FÍSICA SOBRE A APTIDÃO AERÓBIA, FORÇA MUSCULAR E QUALIDADE DE VIDA EM SOBREVIVENTES DE CÂNCER DE MAMA

Pesquisador: Patricia Chakur Brum

Área Temática:

Versão: 1

CAAE: 80445817.0.0000.5391

Instituição Proponente: UNIVERSIDADE DE SAO PAULO

Patrocinador Principal: Financiamento Próprio

DADOS DO PARECER

Número do Parecer: 2.441.435

Apresentação do Projeto:

O projeto visa investigar se um programa de atividade física supervisionado e não estruturado apresenta vantagens potenciais em relação às demais modalidades já
investigadas, reunindo benefícios típicos de uma atividade supervisionada (maior segurança, maior aderência, maior motivação) com as vantagens de uma atividade não estruturada (maior exequibilidade, menor controle, maior conforto, menor custo, maior acessibilidade), promovendo benefícios superiores em qualidade de vida em pacientes sobreviventes de câncer de mama.

Objetivo da Pesquisa:

Objetivo Primário: Comparar os efeitos da atividade física estruturada e supervisionada, atividade física não estruturada e supervisionada e atividade física não estruturada não supervisionada sobre aptidão aeróbia, força muscular e qualidade de vida em pacientes sobreviventes de neoplasia de mama.

Objetivo Secundário:Comparar os efeitos de diferentes modalidades de atividade física sobre A)Desfechos primários: 1.Consumo pico de oxigênio (VO2pico); 2.Força de
preensão manual; 3.Capacidade Funcional de membros inferiores; 4.Qualidade de vida.B)Desfechos secundários: 1.Fadiga; 2.Presença de linfedema; 3.Atividade física diária; 4.Auto imagem; 5.Dor. Composição corporal; 7. Marcadores imunológicos;8. Balanço autonômico.

Avaliação dos Riscos e Benefícios:

Como benefícios ofereceremos a todas as voluntárias orientação para atividade física pós término da pesquisa. Serão oferecidas palestras sobre saúde, qualidade de vida e atividade física pelos pesquisadores.

O risco é baixo, pois será realizada a Coleta e sangue das pacientes.

Comentários e Considerações sobre a Pesquisa:

A pesquisa tem um caráter e função social, auxiliando as pacientes, que tiveram câncer, a terem melhor qualidade de vida. A pesquisa está muito bem delineada e as metodologias usadas bem descritas.

Considerações sobre os Termos de apresentação obrigatória:

O TCLE está bem apresentado, com linguagem adequada para ser entendida pelas participantes do projeto

Recomendações:

Pode ser aprovado

Conclusões ou Pendências e Lista de Inadequações:

O projeto pode ser aprovado. Não apresenta pendências.

Considerações Finais a critério do CEP:

Este parecer foi elaborado baseado nos documentos abaixo relacionados:

Tipo Documento	Arquivo	Postagem	Autor	Situação
Informações Básicas do Projeto	PB_INFORMAÇÕES_BÁSICAS_DO_PROJETO_1039284.pdf	27/11/2017 16:19:37		Aceito
TCLE / Termos de Assentimento / Justificativa de Ausência	teclerem ama.doc	27/11/2017 16:18:50	Patricia Chakur Brum	Aceito
Projeto Detalhado / Brochura Investigador	remama.pdf	27/11/2017 16:18:33	Patricia Chakur Brum	Aceito
Folha de Rosto	scan.pdf	27/11/2017 16:11:51	Patricia Chakur Brum	Aceito

Situação do Parecer:

Endereço: Av. Profº Mello Moraes, 65
Bairro: Cidade Universitária **CEP:** 05.508-030
UF: SP **Município:** SAO PAULO
Telefone: (11)3091-3097 **Fax:** (11)3812-4141 **E-mail:** cep39@usp.br

Aprovado

Necessita Apreciação da CONEP:
Não

SAO PAULO, 15 de Dezembro de 2017

Assinado por:
Edilamar Menezes de Oliveira
(Coordenador)

Endereço: Av. Profº Mello Moraes, 65
Bairro: Cidade Universitária **CEP:** 05.508-030
UF: SP **Município:** SAO PAULO
Telefone: (11)3091-3097 **Fax:** (11)3812-4141 **E-mail:** cep39@usp.br
