# Supplementary material for: The influence of a supervised group exercise intervention combined with active lifestyle recommendations on breast cancer survivors’ health, physical functioning, and quality of life indices: study protocol for a randomized and controlled trial
Source: Trials. 2021 Dec 18;22:934. doi: 10.1186/s13063-021-05843-z (PMC8684206; doi:10.1186/s13063-021-05843-z)
Supplement: Supplementary file 3 — Additional file 3. [file 13063_2021_5843_MOESM3_ESM.pdf]

Technical Report from the Research Ethics Committee

RESEARCH PROJECT INFORMATION

Research title: EFFECTS OF DIFFERENT MODALITIES OF PHYSICAL ACTIVITY ON AEROBIC FITNESS, MUSCLE STRENGTH AND QUALITY OF LIFE IN BREAST CANCER SURVIVORS

Principal investigator (PI): Patricia Chakur Brum

Thematic Area:

Version: 1

CAAE number: 80445817.0.0000.5391

Proponent Institution: UNIVERSITY OF SAO PAULO

Main Sponsor: Self-funded project

TECHNICAL REPORT INFORMATION

Report number: 2.441.435

Project description:

The project aims to investigate whether a supervised and unstructured physical activity program has potential advantages over other modalities already investigated, bringing together typical benefits of a supervised activity (greater safety, greater adherence, greater motivation) with the advantages of an unstructured activity (greater feasibility, less control, greater comfort, lower cost, greater accessibility), promoting superior benefits in quality of life in breast cancer survivors.

Research objectives:

Primary objective: Compare the effects of structured and supervised physical activity, unstructured and supervised physical activity and unstructured unstructured physical activity on aerobic fitness, muscle strength and quality of life in breast cancer surviving patients.

Secondary Objective: Compare the effects of different types of physical activity on:

A) Primary outcomes:

1. Peak oxygen consumption (VO_2 peak),

2. Handgrip strength,
3. Functional capacity of lower limbs,
4. Quality of life.

B) Secondary outcomes:
1. Fatigue,
2. Presence of lymphedema,
3. Daily physical activity,
4. Self-image,
5. Pain,
6. Body composition,
7. Immunological markers,
8. Autonomic balance.

Assessment of risks and benefits:

Benefits of this study include offering guidance for physical activity to all volunteers after the end of the research. Talks on health, quality of life and physical activity will be delivered by researchers. The risk is low, as the blood sampling of the patients will be performed.

Comments and considerations on this research:

The present research plays a social role once it contributes to patients with and beyond breast cancer to reach improved quality of life. The study is very well designed, and the methods are described precisely.

Mandatory documents submission:

The informed consent form is well described, with appropriate language to be understood by the study participants.

Recommendation:

The present project is approved.

Conclusions or list of pending and inadequacies:

The project is approved with no pending issues.

Final Considerations after appreciation of the Research ethical committee:

The current technical report was elaborated based on the following documents:

Document	File	Uploaded in	Author	Status
Basic information	PB_INFORMAÇÕES_BÁSICAS_DO_P	27/11/2017		Accepted
Consent form/absence justificative	tecleremama.doc	27/11/2017 16:18:50	Patricia Chakur Brum	Accepted
Detailed project version/ Booklet	remama.pdf	27/11/2017 16:18:33	Patricia Chakur Brum	Accepted
Cover sheet	scan.pdf	27/11/2017	Patricia Chakur Brum	Accepted

Status of the present project:

Approved.

Need of appreciation by CONEP:

Further appreciation is not required.

SAO PAULO, 15th of December of 2017

Signed by Edilamar Menezes de Oliveira

(Coordinator)
