# Supplementary material for: The influence of a supervised group exercise intervention combined with active lifestyle recommendations on breast cancer survivors’ health, physical functioning, and quality of life indices: study protocol for a randomized and controlled trial
Source: Trials. 2021 Dec 18;22:934. doi: 10.1186/s13063-021-05843-z (PMC8684206; doi:10.1186/s13063-021-05843-z)
Supplement: Supplementary file 4 — Additional file 4. [file 13063_2021_5843_MOESM4_ESM.pdf]

PARECER CONSUBSTANCIADO DO CEP

Elaborado pela Instituição Coparticipante

DADOS DO PROJETO DE PESQUISA

Título da Pesquisa: EFEITOS DE DIFERENTES MODALIDADES DE ATIVIDADE FÍSICA SOBRE A APTIDÃO AERÓBIA, FORÇA MUSCULAR E QUALIDADE DE VIDA EM SOBREVIVENTES DE CÂNCER DE MAMA

Pesquisador: Patricia Chakur Brum

Área Temática:

Versão: 2

CAAE: 80445817.0.3001.0065

Instituição Proponente: FUNDACAO FACULDADE DE MEDICINA

Patrocinador Principal: Financiamento Próprio

DADOS DO PARECER

Número do Parecer: 2.836.680

Apresentação do Projeto:

O presente projeto visa analisar se um programa de atividade física supervisionado e não estruturado apresenta vantagens potenciais em relação às demais modalidades já
investigadas, reunindo benefícios típicos de uma atividade supervisionada (maior segurança, maior aderência, maior motivação) com as vantagens de uma atividade não estruturada (maior exequibilidade, menor controle, maior conforto, menor custo, maior acessibilidade), promovendo benefícios superiores em qualidade de vida em pacientes sobreviventes de câncer de mama.

Objetivo da Pesquisa:

Comparar os efeitos da atividade física estruturada e supervisionada, atividade física não estruturada e supervisionada e atividade física não estruturada não supervisionada sobre aptidão aeróbia, força muscular e qualidade de vida em pacientes sobreviventes de neoplasia de mama.

Comparar os efeitos de diferentes modalidades de atividade física sobre

A)Desfechos primários:

1.Consumo pico de oxigênio (VO2pico);

2.Força de preensão manual;

3.Capacidade Funcional de membros inferiores;

4.Qualidade de vida.
B)Desfechos secundários:
1.Fadiga;
2.Presença de linfedema;
3.Atividade física diária;
4.Auto imagem;
5.Dor;
6.Composição corporal;
7.Marcadores imunológicos;
8.Balanço autonômico.

Avaliação dos Riscos e Benefícios:

Após a coleta de sangue, uma vermelhidão poderá aparecer no antebraço que irá desaparecer em algumas horas. Após a realização do teste de esforço a voluntária poderá sentir-se cansada, mas será avaliada e acompanhada por um médico, permanecendo monitorizada até que aconteça sua total recuperação. O teste de função cardiopulmonar e os procedimentos de coleta de sangue serão realizados por médico devidamente habilitado

Comentários e Considerações sobre a Pesquisa:

Estudo 1 - Atividade não estruturada e supervisionada (treinamento de remo coletivo) - NS x Atividade não estruturada e não supervisionada (cartilha informativa- padrão de saúde pública recomendada pela OMS e ANS-Ministério da Saúde) - NN
Estudo 2 - Atividade não Estruturada e supervisionada (treinamento de remo coletivo) - NS x Atividade física estruturada e supervisionada (padrão ouro- recomendada pelo ACSM) - ES

Considerações sobre os Termos de apresentação obrigatória:

TCLE de acordo com a Resolução 466 (corrigido informações sobre CEP-FMUSP, conforme solicitado no parecer anterior).

Sobre a alteração na Plataforma Brasil a respeito da retenção de amostras, pesquisadora justificou que como o Instituto do Câncer do Estado de São Paulo é a instituição coparticipante, não é possível realizar a alteração solicitada no momento, porém compromete-se a enviar emenda ao ao

CEP da instituição principal (EEFEUSP) com a alteração solicitada.

Conclusões ou Pendências e Lista de Inadequações:

Após a aprovação do projeto pelo CEP-FMUSP, submeter uma emenda ao
CEP da instituição principal (EEFEUSP) com a alteração no item da Plataforma Brasil sobre retenção de amostras.

Considerações Finais a critério do CEP:

Este parecer foi elaborado baseado nos documentos abaixo relacionados:

Tipo Documento	Arquivo	Postagem	Autor	Situação
Informações Básicas do Projeto	PB_INFORMAÇÕES_BÁSICAS_DO_PROJETO_1175073.pdf	10/08/2018 12:57:05		Aceito
Declaração de Pesquisadores	Resposta_parecer_CEPFMUSP.pdf	10/08/2018 12:56:12	Patricia Chakur Brum	Aceito
TCLE / Termos de Assentimento / Justificativa de Ausência	TECLE_padrao_PlataformaCEP.doc	10/08/2018 12:54:52	Patricia Chakur Brum	Aceito
Outros	Parecer_ICESP.pdf	04/07/2018 16:42:07	Patricia Chakur Brum	Aceito
TCLE / Termos de Assentimento / Justificativa de Ausência	tecleremama.doc	27/11/2017 16:18:50	Patricia Chakur Brum	Aceito
Projeto Detalhado / Brochura Investigador	remama.pdf	27/11/2017 16:18:33	Patricia Chakur Brum	Aceito

Situação do Parecer:

Aprovado

Necessita Apreciação da CONEP:

Não

SAO PAULO, 22 de Agosto de 2018

Assinado por:
Antonio de Padua Mansur
(Coordenador)

Endereço: DOUTOR ARNALDO 251 21º andar sala 36
Bairro: PACAEMBU **CEP:** 01.246-903
UF: SP **Município:** SAO PAULO
Telefone: (11)3893-4401 **E-mail:** cep.fm@usp.br
