# Supplementary material for: The influence of a supervised group exercise intervention combined with active lifestyle recommendations on breast cancer survivors’ health, physical functioning, and quality of life indices: study protocol for a randomized and controlled trial
Source: Trials. 2021 Dec 18;22:934. doi: 10.1186/s13063-021-05843-z (PMC8684206; doi:10.1186/s13063-021-05843-z)
Supplement: Supplementary file 5 — Additional file 5. [file 13063_2021_5843_MOESM5_ESM.pdf]

TECHNICAL REPORT FROM THE RESEARCH ETHICS COMMITTEE

Elaborated by the co-participant Institution

RESEARCH PROJECT INFORMATION

Research title: EFFECTS OF DIFFERENT MODALITIES OF PHYSICAL ACTIVITY ON AEROBIC FITNESS, MUSCLE STRENGTH AND QUALITY OF LIFE IN BREAST CANCER SURVIVORS.

Principal investigator (PI): Patricia Chakur Brum.

Thematic area:

Version: 2

CAAE: 80445817.0.3001.0065

Proponent Institution: FUNDACAO FACULDADE DE MEDICINA.

Main Sponsor: Self-funded project.

TECHNICAL REPORT INFORMATION

Technical report number: 2.836.680

This project aims to analyze whether a supervised and unstructured physical activity program has potential advantages over other modalities already investigated, gathering typical benefits of a supervised activity (greater security, greater adherence, greater motivation) with the advantages of an unstructured activity (greater feasibility, less control, greater comfort, lower cost, greater accessibility), promoting superior benefits in quality of life in breast cancer survivors.

Research Objective:

Compare the effects of structured and supervised physical activity, unstructured and supervised physical activity and unstructured physical activity on aerobic fitness, muscle strength and quality of life in breast cancer surviving patients.

Compare the effects of different types of physical activity on:

A) Primary outcomes:

1. Peak oxygen consumption (VO_2 peak),
2. Handgrip strength,
3. Functional capacity of lower limbs,
4. Quality of life.

B) Secondary outcomes:

1. Fatigue,
2. Presence of lymphedema,
3. Daily physical activity,
4. Self image,
5. Pain,
6. Body composition,
7. Immunological markers,
8. Autonomic balance.

Assessment of risks and benefits:

After blood collection, temporary redness may appear in the volunteer forearm. After carrying out the stress test, participants may feel tired; however, they will be monitored by a doctor during the test and until full recovery. The cardiopulmonary function test and blood collection procedures will be performed by a qualified physician.

Research Comments and Considerations:

Study 1 - Unstructured and supervised activity (collective rowing training) - NS x Unstructured and unsupervised activity (information booklet-standard of public health recommended by WHO and ANS-Ministry of Health) – NN.

Study 2 - Unstructured and supervised activity (collective rowing training) - NS x Structured and supervised physical activity (gold standard - recommended by ACSM) – ES.

Mandatory submission terms considerations:

The consent form is in accordance with the resolution 466 (rectified information on Commission of Ethics in Research in Human Beings of the USP Medical School (CEP-FMUSP), as requested in the previous technical report).

As the Cancer Institute of the State of Sao Paulo (ICESP) is the co-participant institution of this study, the researcher stated that it is not possible to carry out alteration regarding the "retention of samples" item on Plataforma Brasil website. However, she agreed to forward an amendment to the Research Ethics Committee of the USP School of Physical Education and Sports (EEFEUSP) requesting this change to be done.

Technical report number: 2.836.680

Conclusions or Pending and List of Inadequacies:

After the project has been approved by CEP-FMUSP, an amendment must be submitted to the Research Ethics Committee of the main institution (EEFEUSP), including the change in the item regarding "sample retention" on the Plataforma Brasil website.

Final Considerations after appreciation of the Research ethical committee:

The current technical report was elaborated based on the following documents:

Document	File	Uploaded in	Author	Status
Project basic information	PB_INFORMAÇÕES_BÁSICAS_DO_PROJETO_1175073.pdf	10/08/2018 12:57:05		Accepted
Researchers statement	Resposta_parecer_CEPFMUSP.pdf	10/08/2018 12:56:12	Patricia Chakur Brum	Accepted
Consent form/absence justification form	TECLE_padrao_PlataformaCEP.doc	10/08/2018 12:54:52	Patricia Chakur Brum	Accepted
Others	Parecer_ICESP.pdf	04/07/2018 16:42:07	Patricia Chakur Brum	Accepted
Consent form/absence justification form	tecleremama.doc	27/11/2017 16:18:50	Patricia Chakur Brum	Accepted
Detailed research project/ booklet/ Investigator	remama.pdf	27/11/2017 16:18:33	Patricia Chakur Brum	Accepted

Technical report status:

Approved.

Need of analysis by CONEP:

Further appreciation is not required.

SAO PAULO, 22nd of August of 2018

Signed by:

Antonio de Padua Mansur

(Coordinator)
