# Supplementary material for: The influence of a supervised group exercise intervention combined with active lifestyle recommendations on breast cancer survivors’ health, physical functioning, and quality of life indices: study protocol for a randomized and controlled trial
Source: Trials. 2021 Dec 18;22:934. doi: 10.1186/s13063-021-05843-z (PMC8684206; doi:10.1186/s13063-021-05843-z)
Supplement: Supplementary file 7 — Additional file 7. [file 13063_2021_5843_MOESM7_ESM.pdf]

FAPESP SAGe

Processo

Identificação do Processo

**Número do Processo**	2020/12514-2 - Projeto de Pesquisa - Regular
**Situação**	Em Execução
**Grupo de Financiamento**	Auxílio à Pesquisa
**Linha de Fomento**	Programas Regulares / Auxílios a Pesquisa / Projeto de Pesquisa / Projeto de Pesquisa - Regular - Fluxo Contínuo
**Beneficiário**	Patricia Chakur Brum
**Responsável**	Patricia Chakur Brum
**Data Início**	01/02/2022
**Duração**	24 mês(es)

**Instituição de Pesquisa/Empresa**	Escola de Educação Física e Esporte/EEFE/USP
**Departamento**	Biodinâmica do Movimento do Corpo Humano
**Data de Abertura**	18/09/2020
**Adesão a um programa**	Não
**Nome do Programa**	
**Objetivos da pesquisa correlacionados com o Programa da FAPESP**	

Projeto - Identificação

Título em Português

Influência do exercício físico supervisionado e recomendações para mudança no estilo de vida sobre a saúde, aptidão física e qualidade de vida de sobreviventes de câncer de mama: ensaio clínico controlado e randomizado

Título em Inglês

The influence of a supervised group exercise intervention combined with active lifestyle recommendations on breast cancer survivors' health, physical functioning, and quality of life indices: a randomized and controlled trial

Classificação

**Grande Área**	Ciências da Saúde
**Área**	Educação Física
**Sub-área**	Outra Subárea Educação Física
**Especialidade**	Fisiologia do exercício

**Palavras-chave**	Breast Neoplasms, Canoeing, Exercise,, Life Quality, motivation, physical fitness

Projeto - Instituições

Instituição de Pesquisa/Empresa Principal

**Nome**	Escola de Educação Física e Esporte/EEFE/USP

Projeto - Pessoas Envolvidas

Equipe

Nome	Função	Horas Semanais Dedicadas ao Projeto	Vigência	Vínculo Principal
Patricia Chakur Brum	Pesquisador Responsável	15	01/02/2022 a 31/01/2024	Escola de Educação Física e Esporte/EEFE/USP
Carlos Ugrinowitsch	Pesquisador	2	01/02/2022 a	Escola de Educação Física e

	Associado		31/01/2024	Esporte/EEFE/USP
Cláudia Lúcia de Moraes Forjaz	Pesquisador Associado	2	01/02/2022 a 31/01/2024	Escola de Educação Física e Esporte/EEFE/USP
Sarah Milani de Moraes Leandrini	Apoio Técnico	20	01/02/2022 a 31/01/2024	Escola de Educação Física e Esporte/EEFE/USP

* Com Benefício Complementar

Projeto - Descrição

Resumo em Portugês

A maioria dos pacientes com câncer, em tratamento ativo ou não, são sedentários, apesar das evidências científicas e clínicas dos benefícios do exercício físico e da atividade física, como melhora da qualidade de vida e dos sintomas da doença, redução da reincidência do câncer e melhora da sobrevida geral. Estudos demonstraram que tanto os programas de exercícios físicos supervisionados quanto os de atividades físicas não supervisionados apresentam baixa adesão e seus benefícios são limitados em longo prazo entre os sobreviventes de câncer. Portanto, intervenções voltadas para o aumento dos níveis de atividade física têm relevância clínica e psicológica. O presente estudo examinará a viabilidade e eficácia de uma intervenção que combina exercícios em grupo supervisionados com recomendações de estilo de vida ativo, analisando seus efeitos clínicos, psicológicos, fisiológicos, funcionais e imunológicos em sobreviventes do câncer de mama. Mulheres de 35 a 75 anos que concluíram quimioterapia, radioterapia e cirurgia para câncer de mama serão recrutadas no Instituto do Câncer do Estado de São Paulo (ICESP) e participarão de um ensaio clínico de 16 semanas, grupo paralelo, randomizado e controlado. Elas receberão um livreto com recomendações para alcançar um estilo de vida fisicamente ativo, aumentando o movimento diário geral e realizando pelo menos 150 min/semana de exercícios estruturados. Em seguida, elas serão randomizadas em dois grupos: a) o grupo supervisionado participará de duas sessões/semana de exercícios em grupo de canoagem; b) o grupo não supervisionado deverá atividade física por qualquer meio, como deslocamento ativo, atividades diárias ou exercícios domiciliares conforme recomendado no libreto. Os desfechos primários incluem capacidade aeróbia, atividade física, funcionamento físico e qualidade de vida auto-relatada. Os desfechos secundários são fadiga, presença de linfedema, composição corporal, função imunológica, adesão às diretrizes de atividade física e percepção da autoimagem. Os resultados devem contribuir para o avanço do conhecimento sobre o impacto de uma intervenção de exercícios físicos supervisionados e realizados em grupo para melhorar os aspectos relacionados à saúde, aptidão física e qualidade de vida em mulheres sobreviventes ao câncer de mama.

Resumo em Inglês

Most cancer patients, under active treatment or not, are sedentary, despite increasing scientific and clinical understanding of the benefits that exercise and physical activity can have, such as improving quality of life, limiting disease symptoms, decreasing cancer recurrence, and increasing overall survival. Studies have shown that both supervised exercise and unsupervised physical activity programs have low adherence and limited long-term benefits among cancer survivors. Therefore, interventions focused on increasing physical activity levels have clinical and psychological relevance. The present study will examine the feasibility and efficacy of an intervention that combines supervised group exercise with active lifestyle recommendations, analyzing its clinical, psychological, physiological, functional, and immunological effects in breast cancer survivors. Women aged 35-75 years who have completed chemotherapy, radiotherapy and surgery for breast cancer will be recruited from the Cancer Institute of the State of Sao Paulo (ICESP) and take part in a 16-week, parallel-group, randomized and controlled trial. They will receive a booklet with recommendations for achieving a physically active lifestyle by increasing overall daily movement and undertaking at least 150 min/week of structured exercise. Then, they will be randomized into two groups: the supervised group will take part in two canoeing group exercise sessions every week; and the unsupervised group will increase their overall physical activity level by any means, such as active commuting, daily activities or home-based exercise. Primary outcomes include aerobic capacity, physical activity, physical functioning, and self-reported quality of life. Secondary outcomes are fatigue, presence of lymphedema, body composition, immune function, adherence to physical activity guidelines, and perceptions of self-image. Results should contribute to advance knowledge on the impact of a supervised group exercise intervention to improve aspects related to health, physical functioning, and quality of life in female breast cancer survivors.

Objetivos

The aim of the study is to assess the impact of a 16-week supervised group exercise intervention, combined with physical activity recommendations delivered via a booklet, on overall physical activity, physical functioning, and quality of life among breast cancer survivors. The primary outcomes include, aerobic capacity (assessed via directly measured peak oxygen consumption, or VO2), physical activity (assessed via accelerometry and questionnaire), physical functioning (assessed via muscle strength, balance, and agility) and self-reported quality of life. Secondary outcomes are fatigue, presence of lymphedema, body composition, immune function, adherence to physical activity guidelines, and perceptions of self-image.

Resultados Previstos

The group exercise intervention combined with physical activity recommendations will display a superior effect on aerobic capacity, physical and immune functioning, physical activity levels, and self-reported quality of life. These results will be accompanied by a higher adherence to physical activity guidelines, which is crucial for sustaining an active lifestyle leading to improvements in global health in female breast cancer survivors.

O projeto poderá obter resultados que justifiquem a solicitação de patente?

Não

R$ / US$ - Orçamento

Orçamento

Benefícios	Valor (R$)	Valor (US$)

Capital		
Material Permanente	14.090,00	4.019,70
Custeio		
Despesas de Transporte	3.326,40	0,00
Diárias	0,00	0,00
Material de Consumo	20.734,00	10.294,00
Serviços de Terceiros	1.652,00	2.195,00
Reserva Técnica - Benefícios Complementares	0,00	0,00
Reserva Técnica - Custo de Infraestrutura Direta do Projeto	20.085,29	0,00
Provisão para Importação	0,00	2.476,30
TOTAL	59.887,69	18.985,00

Quotas de Bolsa

Modalidade / Nível	Carga Horária	Duração (Meses)	Quantidade

Nenhuma quota solicitada.

R$ / US$ - Orçamento - Detalhamento

Material Permanente - Importado

**Origem**	Exterior
**Quantidade**	12
**Classificação**	Acelerômetro
**Descrição**	Acelerômetro GT9X
**Fabricado no Brasil**	Não
**Moeda de Origem**	US$
**Valor Unitário**	275,00
**Taxa de Câmbio (US$)**	1,0000000
**Valor Total**	3.300,00
**Justificativa**	Os acelerômetros serão utilizados para realizar as medidas de níveis de atividade física das voluntárias, gerando dados de tempo despendido em diferentes níveis de atividade física (leve, moderado, intenso) e tempo sedentário.

Acessório

**Origem**	Exterior
**Quantidade**	6
**Descrição**	Base com porta USB
**Fabricado no Brasil**	Não
**Moeda de Origem**	US$
**Valor Unitário**	42,00
**Taxa de Câmbio (US$)**	1,0000000
**Valor Total**	252,00
**Justificativa**	As bases são necessárias para conectar os acelerômetros ao computador, iniciá-los, baixar os dados e carregar a bateria.

Acessório

**Origem**	Exterior
**Quantidade**	12
**Descrição**	Clipe de segurança
**Fabricado no Brasil**	Não
**Moeda de Origem**	US$
**Valor Unitário**	12,00
**Taxa de Câmbio (US$)**	1,0000000
**Valor Total**	144,00
**Justificativa**	Os clipes são indispensáveis para prender os acelerômetros às cintas que as voluntárias utilizarão na cintura.

Acessório

**Origem**	Exterior

**Quantidade**	6
**Descrição**	Pulseira
**Fabricado no Brasil**	Não
**Moeda de Origem**	US$
**Valor Unitário**	21,95
**Taxa de Câmbio (US$)**	1,0000000
**Valor Total**	131,70
**Justificativa**	As pulseiras são necessárias para manter os acelerômetros bem posicionados no pulso das voluntárias.

Acessório

**Origem**	Exterior
**Quantidade**	8
**Descrição**	Cinta elástica 150cm
**Fabricado no Brasil**	Não
**Moeda de Origem**	US$
**Valor Unitário**	16,00
**Taxa de Câmbio (US$)**	1,0000000
**Valor Total**	128,00
**Justificativa**	As cintas são necessárias para manter o acelerômetro posicionado na cintura das voluntárias.

Acessório

**Origem**	Exterior
**Quantidade**	4
**Descrição**	Cinta elástica 120 cm
**Fabricado no Brasil**	Não
**Moeda de Origem**	US$
**Valor Unitário**	16,00
**Taxa de Câmbio (US$)**	1,0000000
**Valor Total**	64,00
**Justificativa**	As cintas são necessárias para manter o acelerômetro posicionado na cintura das voluntárias.

Acessório

**Origem**	Exterior
**Quantidade**	0
**Descrição**	Contrato de manutenção de atualização
**Fabricado no Brasil**	Não
**Moeda de Origem**	US$
**Valor Unitário**	0,00
**Taxa de Câmbio (US$)**	1,0000000
**Valor Total**	0,00
**Justificativa**	A compra do software é uma licença permanente de uso, porém as atualizações são válidas somente por um ano. Portanto, para ter as atualizações do software pelo período do projeto é necessário comprar mais um ano do contrato de manutenção.

Acessório

**Origem**	Exterior
**Quantidade**	0
**Descrição**	Software Actilife
**Fabricado no Brasil**	Não
**Moeda de Origem**	US$
**Valor Unitário**	0,00
**Taxa de Câmbio (US$)**	1,0000000
**Valor Total**	0,00
**Justificativa**	O software Actilife é necessário para configurar os acelerômetros, baixar e analisar os dados gerados. A licença comprada permite o acesso pleno (iniciar, baixar e analisar os dados) em um computador e o acesso "Lite" (somente iniciar e baixar dados) em cinco computadores.

Material Permanente - Nacional

**Origem**	Brasil

**Quantidade**	10
**Classificação**	Sensor
**Descrição**	Sensor de frequência cardíaca
**Fabricado no Brasil**	Sim
**Valor Unitário**	549,00
**Valor Total**	5.490,00
**Justificativa**	O sensor de frequência cardíaca é necessário para medir a FC das voluntárias durante o treino, fornecendo dados de esforço e para verificar se elas atingem FC máxima e permanecem na FC entre limiares ventilatórios.

Material Permanente - Nacional

**Origem**	Brasil
**Quantidade**	1
**Classificação**	Relógio monitor cardíaco com GPS
**Descrição**	Relógio com monitor cardíaco e GPS
**Fabricado no Brasil**	Sim
**Valor Unitário**	3.700,00
**Valor Total**	3.700,00
**Justificativa**	O relógio será utilizado durante as visitas de coleta de dados para monitorar a variabilidade da frequência cardíaca e também durante os treinos, para registrar dados sobre distância e velocidade percorrida pelo barco.

Material Permanente - Nacional

**Origem**	Brasil
**Quantidade**	2
**Classificação**	Microcomputador portátil e componentes
**Descrição**	Tablet com tela de 10'1, Memória interna 32G interna, Memória RAM 2G
**Fabricado no Brasil**	Sim
**Valor Unitário**	1.700,00
**Valor Total**	3.400,00
**Justificativa**	Os tablets são necessários para verificar o funcionamento dos monitores de frequência cardíaca durante o treinamento, para baixar os dados dos mesmos e aplicar os questionários para as voluntárias. As especificações estão de acordo com as necessárias para compatibilidade com os monitores de frequência cardíaca.

Material Permanente - Nacional

**Origem**	Brasil
**Quantidade**	1
**Classificação**	Velocímetro
**Descrição**	Ciclocomputador com GPS
**Fabricado no Brasil**	Sim
**Valor Unitário**	1.500,00
**Valor Total**	1.500,00
**Justificativa**	O ciclocomputador com GPS é necessário para os treinos de canoagem, fornecendo medidas sobre o tempo de exercício, velocidade e distância percorrida pelos barcos.

Despesas de Transporte - Nacional

**Origem**	Brasil
**Quantidade**	756
**Classificação**	Despesa de Transporte
**Descrição**	Passagem unitária de ônibus/metrô.
**Valor Unitário**	4,40
**Valor Total**	3.326,40
**Justificativa**	Será utilizada no auxílio do deslocamento das voluntárias de sua residência até a Universidade nos dias de coleta de dados e participação no programa de exercício.

Material de Consumo - Importado

**Origem**	Exterior
**Classificação**	Material de Consumo
**Descrição**	Peptídeos
**Moeda de Origem**	US$
**Valor**	4.969,00
**Taxa de Câmbio (US$)**	1,0000000

**Valor Total**	4.969,00
**Justificativa**	Os peptídeos são utilizados como antígenos para estimular as células mononucleares e verificar sua função. Utilizaremos peptídeos controles (virais) e antígenos associados a tumores.

Material de Consumo - Importado

**Origem**	Exterior
**Classificação**	Material de Consumo
**Descrição**	Anticorpos e reagentes
**Moeda de Origem**	US$
**Valor**	2.855,00
**Taxa de Câmbio (US$)**	1,0000000
**Valor Total**	2.855,00
**Justificativa**	Anticorpos e reagentes para cultura celular, isolamento de PBMCs, ELISPOT.

Material de Consumo - Importado

**Origem**	Exterior
**Classificação**	Material de Consumo
**Descrição**	Material plástico consumível
**Moeda de Origem**	US$
**Valor**	2.470,00
**Taxa de Câmbio (US$)**	1,0000000
**Valor Total**	2.470,00
**Justificativa**	placas para cultivo celular, ponteiras para micropipeta, tubos de centrifugação, tubos para isolamento de PBMCs são necessários para desenvolver os experimentos de cultura celular, isolamento de PBMCs, ELISPOT.

Material de Consumo - Nacional

**Origem**	Brasil
**Classificação**	Material de Consumo
**Descrição**	Reagentes para experimentos no laboratório
**Valor**	10.040,00
**Justificativa**	Reagentes necessários para desenvolver os experimentos de cultura celular, isolamento de PBMCs, ELISPOT e outros procedimentos em condições estéreis.

Material de Consumo - Nacional

**Origem**	Brasil
**Classificação**	Material de Consumo
**Descrição**	Material consumível para experimentos no laboratório
**Valor**	7.254,00
**Justificativa**	Materiais necessários para desenvolver os experimentos de cultura celular, isolamento de PBMCs, ELISPOT e outros procedimentos em condições estéreis: Pipeta Pasteur plástica, Tubos Cônicos, Pipetas Sorológicas, Microtubos, Filtros de seringa, Seringa, Reservatório de reagente estéril, Luvas de procedimento

Material de Consumo - Nacional

**Origem**	Brasil
**Classificação**	Material de Consumo
**Descrição**	Material para coleta de sangue
**Valor**	1.860,00
**Justificativa**	Material necessário para coletar sangue das voluntárias: Compressa de Gaze Hidrofila e Álcool 70% para fazer assepsia, Curativo redondo, Garrote, Agulha e Adaptador para coleta a vácuo, Seringa, Escalpe, Tubos Vacutainer BD, Luvas de procedimento, Coletor de perfurocortantes contaminados, Saco para lixo contaminante.

Material de Consumo - Nacional

**Origem**	Brasil
**Classificação**	Material de Consumo
**Descrição**	Materiais para teste ergoespirométrico
**Valor**	1.580,00
**Justificativa**	Os materiais são necessários para o teste ergoespirométrico: Eletrodos e Gel condutor para monitorar FC, Compressa de Gaze Hidrofila e Álcool 70% para fazer assepsia das

voluntárias, Esparadrapo para fixar eletrodos, Lençol de Papel para forrar a maca, Máscara de neoprene para ficar o respirador.

Serviços de Terceiros - Importado

**Origem**	Exterior
**Quantidade**	1
**Classificação**	Serviço de Terceiros
**Descrição**	Software Actilife
**Moeda de Origem**	US$
**Valor Unitário**	2.195,00
**Taxa de Câmbio (US$)**	1,0000000
**Valor Total**	2.195,00
**Justificativa**	O software Actilife é necessário para configurar os acelerômetros, baixar e analisar os dados gerados. A licença comprada permite o acesso pleno (iniciar, baixar e analisar os dados) em um computador e o acesso "Lite" (somente iniciar e baixar dados) em cinco computadores.

Serviços de Terceiros - Nacional

**Origem**	Brasil
**Quantidade**	72
**Classificação**	Serviço de Terceiros
**Descrição**	Exames de sangue (Hemograma completo, Colesterol e frações, Glicemia, Hemoglobina Glicada)
**Valor Unitário**	16,00
**Valor Total**	1.152,00
**Justificativa**	As análises de sangue das voluntárias são essenciais para verificar estado geral de saúde e fatores de risco associados às consequências do câncer e tratamentos. Tais exames serão realizados nos momentos pré e pós intervenção.

Serviços de Terceiros - Nacional

**Origem**	Brasil
**Quantidade**	10
**Classificação**	Serviço de Terceiros
**Descrição**	Leitor de placas para ELISPOT.
**Valor Unitário**	50,00
**Valor Total**	500,00
**Justificativa**	Valor de custo estimado para a hora de utilização do leitor, essencial para realizar a leitura das placas de ELISPOT e analisar a função das células imunológicas.

Reserva Técnica - Custo de Infraestrutura Direta do Projeto

**Percentual para Reserva Técnica (País)**	15,00 %
**Percentual para Reserva Técnica (Exterior)**	15,00 %
**Dólar FAPESP**	5,70
**Valor Aumentado**	0,00
**Valor Diminuído**	0,00
**Valor da Reserva Técnica (R$)**	20.085,29
**Valor da Reserva Técnica (US$)**	0,00

Provisão para Importação

**Percentual para Provisão para Importação**	15,00 %
**Valor da Provisão para Importação (US$)**	2.476,30

R$ / US$ - Outras Fontes

Outras Fontes

Nenhuma outra fonte encontrada.

Documentos

Download de Todos os Documentos

1.1 Documentos Anexados na Proposta Atual (Proposta Inicial submetida em 18/09/2020)

Tipo de Documento	Etapa Exigida	Arquivo	Data de Anexação	Arquivo Convertido
Anexo II: Informação aprovada pela Instituição Sede sobre a infraestrutura institucional	Análise	E-mail Infraestrutura Institucional-Diretor.pdf	21/09/2020	↓
Descrição das atividades desenvolvidas pela equipe	Análise	Group Activity Description.pdf	18/09/2020	↓
Manifestação do Dirigente da Instituição Sede	Análise	Email com manifestacao do dirigente-diligencia.pdf	21/09/2020	↓
Orçamentos dos fornecedores/representantes autorizados	Análise	Proforma e Exclusividade Actigraph.zip	04/09/2020	↓
Parque de equipamentos	Análise	Parque de equipamentos Remama.pdf	17/09/2020	↓
Plano de gestão de dados	Análise	The_influence_of_a_supervised_group_exercise_interventio.pdf	16/09/2020	↓
Planos de atividades individuais para cada bolsa de treinamento técnico e/ou participação	Análise	Não se Aplica		
Projeto de pesquisa (auxílio)	Análise	Projeto Remama APR FAPESP_Versa_o definitiva.pdf	17/09/2020	↓
Resultados de auxílios anteriores	Análise	Resultados de auxi_lios anteriores 09-2020 final certo.pdf	18/09/2020	↓
Súmula curricular de cada um dos pesquisadores associados	Análise	Su_mula Pesq.associados.pdf	17/09/2020	↓
Súmula curricular do beneficiário	Análise	Su_mula Curricular Patri_cia Brum- Sep 2020.pdf	16/09/2020	↓

1.2 Outros Documentos Anexados na Proposta Atual (Proposta Inicial submetida em 18/09/2020)

Arquivo	Data de Anexação	Arquivo Convertido
James_Turner_Letter_2020-08-29.pdf	16/09/2020	↓
Sumulas Claudia e Carlos PAs.pdf	21/09/2020	↓

1.3 Documentos Anexados pela FAPESP na Proposta Atual (Proposta Inicial submetida em 18/09/2020)

Nenhum documento associado.

Observações / Manifestações

Observações

Histórico de Eventos

Histórico de Eventos

Descrição	Data
Assinatura da FAPESP Registrada - Contrato Inicial	28/06/2021
Assinatura do Outorgado Registrada - Contrato Inicial	28/06/2021
Análise da Minuta Concluída - Contrato Inicial	11/06/2021
Preparação da Minuta Concluída - Contrato Inicial	21/05/2021
Verificação da habilitação com resultado "Habilitado" - Contrato Inicial	21/05/2021
Aceite da Concessão com resultado "Aprovado"	20/05/2021
Decisão do CTA - SM 001 - Alteração de Vigência	11/05/2021
Emissão de Despacho Científico Concluída - SM 001 - Alteração de Vigência	10/05/2021
Preparação de Despacho Científico Concluída - SM 001 - Alteração de Vigência	10/05/2021
Despacho Científico Iniciado - SM 001 - Alteração de Vigência	10/05/2021

Recomendação da Coordenação Adjunta Concluída - SM 001 - Alteração de Vigência	10/05/2021
Submissão - SM 001 - Alteração de Vigência	07/05/2021
Aceite da Concessão com resultado "Solicitado Mudança(s)"	07/05/2021
Decisão do CTA - Proposta Inicial	19/03/2021
Emissão de Despacho Científico Concluída - Proposta Inicial	16/03/2021
Preparação de Despacho Científico Concluída - Proposta Inicial	15/03/2021
Pré-Preparação de Despacho Científico Concluída - Proposta Inicial	24/02/2021
Despacho Científico Iniciado - Proposta Inicial	24/02/2021
Recomendação da Coordenação Adjunta Concluída - Proposta Inicial	11/02/2021
Indicação de Assessor ad-hoc Removida - Proposta Inicial	10/01/2021
Parecer de Assessor ad-hoc Emitido - Proposta Inicial	28/12/2020
Solicitação enviada a Assessor ad-hoc para emissão de parecer - Proposta Inicial	02/12/2020
Aprovação da Indicação de Assessor ad-hoc Concluída - Proposta Inicial	23/11/2020
Aprovação da Indicação de Assessor ad-hoc Concluída - Proposta Inicial	23/11/2020
Indicação de Assessor ad-hoc Removida - Proposta Inicial	10/11/2020
Aprovação da Indicação de Assessor ad-hoc Concluída - Proposta Inicial	05/11/2020
Parecer de Assessor ad-hoc Emitido - Proposta Inicial	29/10/2020
Solicitação devolvida pelo Assessor ad-hoc sem emissão de parecer - Proposta Inicial	14/10/2020
Solicitação enviada a Assessor ad-hoc para emissão de parecer - Proposta Inicial	29/09/2020
Solicitação enviada a Assessor ad-hoc para emissão de parecer - Proposta Inicial	29/09/2020
Aprovação da Indicação de Assessor ad-hoc Concluída - Proposta Inicial	28/09/2020
Habilitação Concluída - Proposta Inicial	22/09/2020
Diligência Respondida - Proposta Inicial	21/09/2020
Diligência Aberta - Proposta Inicial	18/09/2020
Habilitação Iniciada - Proposta Inicial	18/09/2020
Submissão da Solicitação - Proposta Inicial	18/09/2020
